# Metabolism-associated danger signal-induced immune response and reverse immune checkpoint-activated CD40^+^ monocyte differentiation

**DOI:** 10.1186/s13045-017-0504-1

**Published:** 2017-07-24

**Authors:** Jin Dai, Pu Fang, Jason Saredy, Hang Xi, Cueto Ramon, William Yang, Eric T. Choi, Yong Ji, Wei Mao, Xiaofeng Yang, Hong Wang

**Affiliations:** 1 Department of Cardiology, The First Affiliated Hospital of Zhejiang Chinese Medical University, 54 Youdian road, Hangzhou, 310006 Zhejiang China; 2 Center for Metabolic Disease Research, Temple University School of Medicine, 3500 N. Broad Street, Philadelphia, PA 19140 USA; 3 Department of Pharmacology, Temple University School of Medicine, 3500 N. Broad Street, Philadelphia, PA 19140 USA; 4 Department of Surgery, Temple University School of Medicine, 3500 N. Broad Street, Philadelphia, PA 19140 USA; 5 Key Laboratory of Cardiovascular and Cerebrovascular Medicine, School of Pharmacy, Nanjing Medical University, Nanjing, 210029 China

**Keywords:** Immune checkpoint, Immune checkpoint, Metabolism-associated danger signal, Metabolic risk factors, Reverse-immune checkpoint, CD40^+^ MC

## Abstract

Adaptive immunity is critical for disease progression and modulates T cell (TC) and antigen-presenting cell (APC) functions. Three signals were initially proposed for adaptive immune activation: signal 1 antigen recognition, signal 2 co-stimulation or co-inhibition, and signal 3 cytokine stimulation. In this article, we propose to term signal 2 as an immune checkpoint, which describes interactions of paired molecules leading to stimulation (stimulatory immune checkpoint) or inhibition (inhibitory immune checkpoint) of an immune response. We classify immune checkpoint into two categories: one-way immune checkpoint for forward signaling towards TC only, and two-way immune checkpoint for both forward and reverse signaling towards TC and APC, respectively. Recently, we and others provided evidence suggesting that metabolic risk factors (RF) activate innate and adaptive immunity, involving the induction of immune checkpoint molecules. We summarize these findings and suggest a novel theory, metabolism-associated danger signal (MADS) recognition, by which metabolic RF activate innate and adaptive immunity. We emphasize that MADS activates the reverse immune checkpoint which leads to APC inflammation in innate and adaptive immunity. Our recent evidence is shown that metabolic RF, such as uremic toxin or hyperhomocysteinemia, induced immune checkpoint molecule CD40 expression in monocytes (MC) and elevated serum soluble CD40 ligand (sCD40L) resulting in CD40^+^ MC differentiation. We propose that CD40^+^ MC is a novel pro-inflammatory MC subset and a reliable biomarker for chronic kidney disease severity. We summarize that CD40:CD40L immune checkpoint can induce TC and APC activation via forward stimulatory, reverse stimulatory, and TC contact-independent immune checkpoints. Finally, we modeled metabolic RF-induced two-way stimulatory immune checkpoint amplification and discussed potential signaling pathways including AP-1, NF-κB, NFAT, STAT, and DNA methylation and their contribution to systemic and tissue inflammation.

**Electronic supplementary material:**

The online version of this article (doi:10.1186/s13045-017-0504-1) contains supplementary material, which is available to authorized users.

## Background

**Fig. 1:**
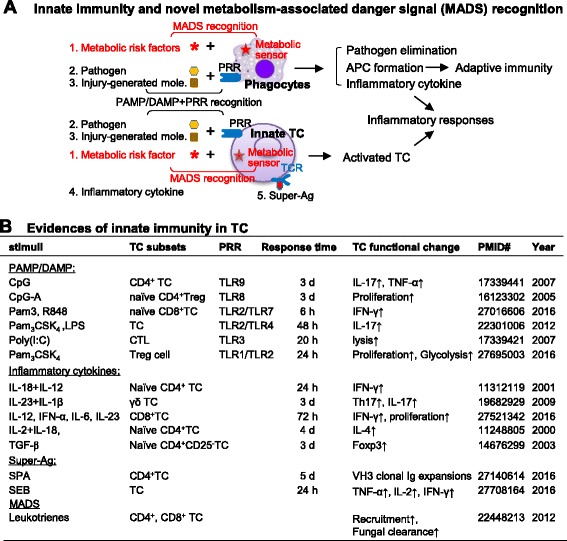
Innate immunity. **a** Innate immunity and novel MADS recognition. The classical innate immune system provides immediate and non-specific defense against pathogen or injury-generated molecules via PAMP/DAMP+PRR recognition in phagocytes and TC. Super Ag, a subset of pathogen toxins, can also bind to a multitude of TCR leading to TC activation. In addition, we propose a novel MADS recognition pathway, which allows metabolic risk factors to activate the innate immunity via responsive metabolic sensors in phagocytes and TC. The activation of innate immunity leads to pathogen elimination and inflammation (APC formation, cytokine generation, and TC activation). **b** Evidences of innate immunity in TC. Stimuli such as PAMP/DAMP, inflammatory cytokines and super Ag activate different subsets of TC and stimulate TC proliferation, inflammatory cytokine production, and phagocytosis. *Words in red* emphasize our newly proposed recognition pattern. Abbreviations: *APC* antigen present cell; *Ag* antigen; *Ab* antibody; *BC* B cell; *BCR* B cell receptor; *CpG* C, a cytosine triphosphate deoxynucleotide; *p* phosphodiester; *G* a guanine triphosphate deoxynucleotide; *CTL* cytotoxic T lymphocytes; *DAMP* danger-associated molecular patterns; *d* days; *Foxp3* forkhead box P3; *h* hours; *IL* interleukin; *IFN* interferon; *LPS* lipopolysaccharide; *MHC* major histocompatibility complex; *MADS* metabolism-associated danger signal; *NLR* NOD (nucleotide-binding and oligomerization domain)-like receptors; *PAMP* pathogen-associated molecular patterns; *PRR* pattern recognition receptor; *Poly(I:C)* polyinosinic-polycytidylic acid; *Pam*_*3*_ *CSK*_*4*_ tripalmitoyl-S-glycero-Cys-(Lys)4; *RF* risk factor; *R848* Imidazoquinoline Resiquimod; *SEB* staphylococcal enterotoxin B; *TC* T cell; *TCR* T cell receptor; *Th17* T helper 17 cell; *TLR* Toll-like receptors; *SPA* staphylococcal protein A; *TNF* tumor necrosis factor; *TGF-β* transforming growth factor beta

The immune system consists of innate and adaptive immunity. The classical innate immune system provides immediate and non-specific defense. It is activated by pathogens via a pathogen-associated molecular pattern (PAMP), which is recognized by pattern recognition receptors (PRR) in phagocytes (Fig. 1). Innate immunity may also be activated in response to injury, which releases a danger-associated molecular pattern (DAMP) also recognized by PRR. These two pathways are summarized as PAMP/DAMP+PRR recognition which leads to pathogen elimination, inflammatory responses, and antigen-presenting cell (APC) formation [1]. Evidence also suggests that the innate immune system targets innate T cells (TC) leading to TC activation [2, 3].

**Fig. 2:**
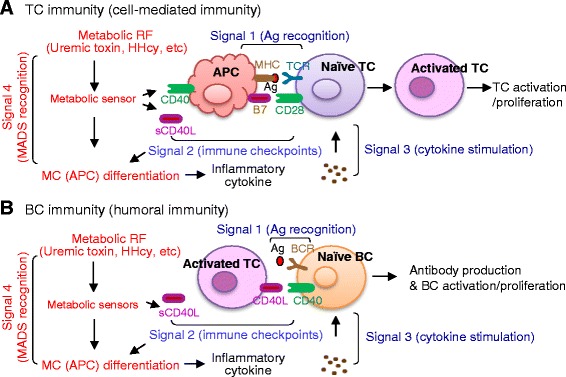
Adaptive immunity with novel signal 4, the metabolic RF recognition. The adaptive immunity is characterized by Ag specificity and immunologic memory leading to TC and BC activation. There are two types of adaptive immunity: TC immunity (cell-mediated immunity) and BC immunity (humoral immunity). Classically, each involves three activating signals. We propose a novel signal 4 (metabolic RF recognition) mediated by metabolic sensor. **a** TC immunity. TC activation involves four distinct signals. In signal 1 (Ag recognition), the Ag peptide is presented by MHC on the APC to Ag-specific TCR on TC. Signal 2 (immune checkpoints) involves ligand and receptor binding on APC and TC. Signal 3 responds to inflammatory cytokine stimulation. The novel signal 4 describes metabolic RF using a metabolic sensor leading to MC (APC) differentiation, inflammatory cytokine production, and the enhancement of signals 2 and 3. **b** BC immunity. BC activation involves Ag binding to BCR (signal 1), ligand and receptor binding (signal 2), cytokine stimulation (signal 3), and metabolic RF recognition (signal 4). *Words in red* emphasize our newly proposed signal. Abbreviations: *APC* antigen present cell, *Ag* antigen, *BC* B cell, *BCR* B cell receptor, *RF* risk factor, *HHcy* hyperhomocysteinemia, *MHC* major histocompatibility complex, *MC* monocyte, *sCD40L* soluble CD40 ligand

Different from innate immunity, adaptive immunity is featured by antigen (Ag) specificity, slow response, immunologic memorization, and low responsive cell ratio (Additional file 1: Table S1) [4]. Adaptive immunity comprises of cell-mediated immunity using TC and B cell (BC) humoral immunity. Each type of adaptive immunity contains three activating signals: (1) Ag recognition, (2) co-stimulation (we termed as immune checkpoint in this article), and (3) cytokine stimulation (Fig. 2). The term of immune checkpoint was initially proposed in 2009 referring to co-inhibitory immune checkpoint for TC suppression [5, 6] and was expanded in 2012 to include co-stimulatory immune checkpoint for TC activation [7]. The concept of immune checkpoint has been extensively studied in recent years and summarized in Table 1. It has become evident that the immune checkpoint plays an important regulatory role in adaptive immunity and determines the fate of the immune cell towards activation or suppression.

**Table 1:**
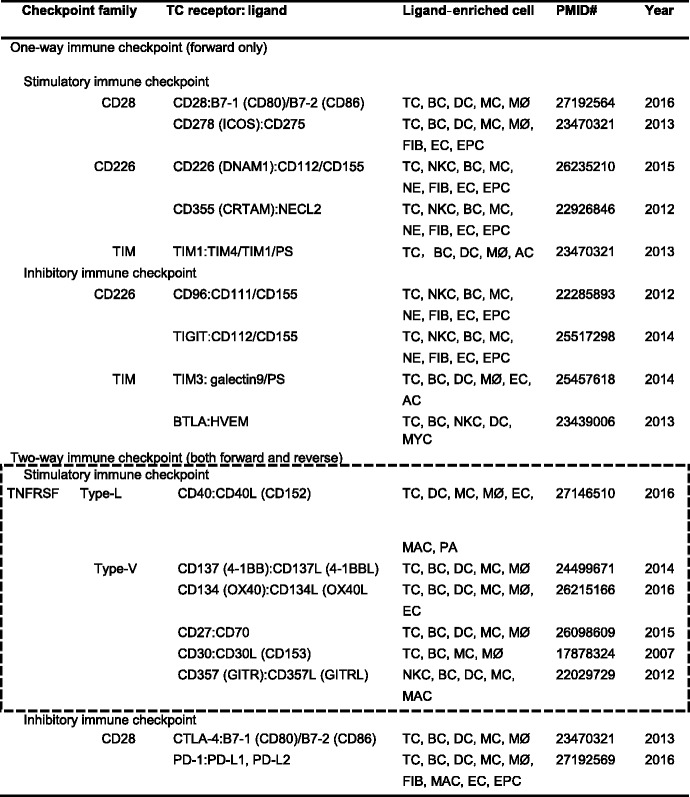
Immune checkpoint families and paired molecules

Immune checkpoints are classified as one-way immune checkpoint and two-way immune checkpoint based on signal 2 direction (forward only or both forward and reverse) and are further divided into stimulatory and inhibitory checkpoints. Listed are a few major immune checkpoint families. For example, in the CD28 family, CTLA-4 receptor binds to ligand B7-1(CD80) or B7-2(CD86) which are enriched in TC, BC, DC, MC, and MØ. The black frame emphasizes the two-way immune checkpoint to be focused. Words in the parentheses are aliases of the receptor and ligands

Abbreviations: *AC* apoptotic cell, *BC* B cell, *BTLA* B and T lymphocyte attenuator, *CTLA-4* cytotoxic T lymphocyte-associated protein 4, *CRTAM* cytotoxic and regulatory T cell molecule, *DC* dendritic cell, *DNAM-1* DNAX accessory molecule-1, *EC* endothelial cell, *EPC* epithelial cell, *FIB* fibroblast, *GITR* glucocorticoid-induced TNFR-related protein, *GITRL* GITR ligand, *HVEM* herpes virus entry mediator, *ICOS* inducible T cell co-stimulator, *MC* monocyte, *MØ* macrophage, *MAC* mast cell, *MYC* myeloid cell, *NKC* natural killer cell, *NE* neuronal, *NECL2* nectin-like protein 2, *PA* platelet, *PS* phosphatidylserine, *PD-1* programmed cell death protein 1, *PD-L* PD ligand, *SMC* smooth muscle cell, *TC* T cell, *TNFSF* tumor necrosis factor superfamily, *TIM* T cell (or transmembrane) immunoglobulin and mucin domain, *TIGIT* T cell immunoreceptor with Ig and ITIM domains

Increased knowledge in immune checkpoints established advances in cancer medicine. For example, immune checkpoint molecule cytotoxic T lymphocyte-associated protein 4 (CTLA-4)-immunoglobulin (Ig) competes with CD28 to bind to CD80/CD86 and causes CTLA-4:CD80/CD86-induced TC suppression [8]. Antibodies against immune checkpoints, CTLA-4 (ipilimumab) and programmed cell death protein 1 (PD-1) (pembrolizumab and nivolumab), block CTLA-4:B7 and PD-1:PD-L1-induced TC suppression and thus enhance TC-dependent immune reaction [9–11]. These antibodies resulted in clinical regression of melanoma, non-small cell lung cancer, and other cancers [9–11]. Immune checkpoint therapy has also proven beneficial for inflammatory diseases such as rheumatoid arthritis and psoriasis using strategies to alleviate inflammation by engaging the inhibitory immune checkpoint [12, 13]. Immune checkpoint therapy for metabolic disease has not yet been realized, but it is an important consideration to balance TC responses and modulate immune checkpoints in contemplating therapies for metabolic disease.

The initial definition of immune checkpoints refers to receptor:ligand reaction towards TC suppression, also referred as co-inhibitory immune checkpoint. The immune checkpoint concept gradually evolved to incorporate co-stimulatory immune checkpoint and the identification of a reverse function of immune checkpoint towards APC [7, 14]. Recent evidence also suggests that metabolic risk factors (RF) can activate the stimulatory immune checkpoint leading to APC-related inflammatory responses [15–19].

We propose a novel metabolism-associated danger signal (MADS) recognition, which promotes reverse stimulatory immune checkpoint leading to APC inflammation in both innate and adaptive immunity systems. MADS refers to intermediates and products of glucose, lipid, amino acid, nucleotide, hormone, and/or chemical metabolism, which can be recognized by the immune system via a metabolic sensor in a receptor-independent fashion.

In this article, we updated the molecular basis regulating innate and adaptive immunity. We proposed two novel nomenclatures, MADS recognition and *reverse immune checkpoint*, and suggested a new theory that MADS recognition regulates innate and adaptive immune response, via metabolic sensor, leading to immune cell activation and inflammation. Information described in this article should provide systemic knowledge and comprehensive insights into our understanding about immune response and immune checkpoints, especially the reverse stimulatory immune checkpoint in diseases.

## Innate immunity recognizes novel MADS and regulates TC activation

### Innate immunity and novel MADS recognition

The innate immune system is activated by pathogens via PAMP+PRR recognition and by injury-generated molecules via DAMP+PRR recognition (Fig. 1a). PRR are receptors presented on all immune cells and somatic cells, which bind to DAMP and PAMP to initiate inflammation [2, 3, 16, 20, 21]. Phagocytes, including macrophage (MØ), monocyte (MC), dendritic cell (DC), neutrophil, and natural killer (NK) cells, are activated by PAMP/DAMP+PRR recognitions which lead to pathogen elimination and inflammatory responses such as APC formation and cytokine generation [1]. Toll-like receptors (TLR) are a key PRR located on the cell surface and endosomes. Nucleotide binding and oligomerization domain-like receptors (NLR) are another important cytosolic-sensing DAMP receptor. In addition, transmembrane C-type lectin (TmCL), retinoid acid-inducible gene I (RIG-I), absent in melanoma 2 (AIM2), and receptor for advanced glycation end products (RAGE) are also characterized as classical DAMP-sensing receptors [22].

We and others provided evidence suggesting that metabolic RF activate innate immune systems leading to inflammatory responses. For example, lipid metabolite ox-LDL promoted NLRP3 inflammasome activation in MØ and foam cell formation [23]. Intermediate amino acid homocysteine (Hcy) induced nucleotide-binding oligomerization domain and leucine-rich repeat and pyrin domain containing protein 3 (NLRP3), causing NLRP3-containing inflammasome assembly, caspase-1 activation, and interleukin (IL)-1β cleavage/activation in EC [16]. Glucose, ceramide, islet amyloid polypeptide, and cholesterol crystals can be sensed by TLR or NLRP3-stimulating NLRP3 inflammasome complex assembly [16, 24, 25]. We [15] and others [26] demonstrated that MADS, such as Hcy or ox-LDL, induced MC activation in the absence of Ag within 48 h. Our data supported the notion that metabolism sensors mediate metabolic RF-induced inflammatory response in the innate immune system (Fig. 1a). Recently, we identified increased Hcy and a reduced ratio of S-adenosylmethionine (SAM)/S-adenosylhomocysteine (SAH), an indicator of cellular methylation, as the metabolic mediator/sensor for pro-inflammatory MC differentiation caused by uremic toxin in chronic kidney disease (CKD) [15].

### Innate immunity in TC

CD4^+^ or CD8^+^ TC, including regulatory TC (Treg), express TLR and is directly involved in innate immunity (Fig. 1b). It is reported that PAMP/DAMP-TLR signaling lead to TC proliferation, inflammatory cytokine production, and glycolysis [2, 3]. Some inflammatory cytokines, such as IL-18, IL-12, IL-1β, IL-23, transforming growth factor (TGF)-β, and interferon (IFN)-α, quickly induced TC subset differentiation and proliferation and IFN-γ, IL-17, and IL-4 secretion in an Ag-independent fashion [6, 27, 28]. Super Ag caused non-specific TC activation and cytokine release [29]. In addition, lipid mediators, such as leukotrienes, are important activators for CD4^+^ and CD8^+^ TC recruitment to the site of infection and control fungal infection [30]. These evidences support the concept of innate immune response in TC via five mechanisms: PAMP/DAMP+PRR recognition, inflammatory cytokines, super Ag, and MADS recognition (Fig. 1).

## Adaptive immunity recognizes MADS and regulates TC/BC activation

The major features of adaptive immunity are Ag specificity and immunologic memory which led to TC and BC activation (Additional file 1: Table S1). It was initially proposed that TC and BC activation involve three signals: signal 1 Ag recognition, signal 2 co-stimulation or co-inhibition, and signal 3 cytokine stimulation (Fig. 2). In this article, we termed signal 2 as the immune checkpoint which is in agreement with Dr. Pardoll’s suggestion in 2012 [7]. We defined immune checkpoint as interactions of paired molecules leading to either stimulatory or inhibitory immune response in TC and BC (other cells as well).

### TC immunity (cell-mediated immunity) (Fig. 2a)

The discovery of TC receptors (TCR) led to defining TC activation signal 1, Ag recognition. Moreover, TC activation signal 2, immune checkpoint, was found to be essential for complete TC activation. For example, CD28 monoclonal antibody administration with simultaneously stimulating TCR leads to complete TC activation [5]. Signal 3, cytokine stimulation, is also involved in TC activation [31]. CD8^+^ TC’s response to virus was shown to be IFN-α dependent. We proposed a novel signal 4 because metabolic RF,, such as uremic toxin and hyperhomocysteinemia (HHcy), activated CD40:CD40L co-stimulatory immune checkpoint and increased serum soluble CD40L (sCD40L) levels [15].

Signal 1 (Ag recognition) is a vital immune process and determines the specificity of TC response. Ag is presented by major histocompatibility complexes (MHC) on the surface of an APC, then engaging with Ag-specific TCR on naïve TC contributing to TC activation/proliferation.

Signal 2 (immune checkpoint) plays a key role in regulating TC activation, differentiation, effector function, and deletion. Signal 2 was initially defined as co-stimulation and expanded to include co-inhibitory pathways [32]. In this article, we propose to term the co-stimulatory and co-inhibitory pathways collectively as the immune checkpoint. Immune checkpoint initially described co-inhibitory signal 2 in Topalian et al.’s papers [33] based on the discovery of T cell function restraint in normal physiologic settings and tumors [34]. This terminology was recently used to describe as a regulatory switch towards either stimulatory or inhibitory pathways [7]. Following Ag recognition or metabolic stimulation, an immune checkpoint ligand on APC binds to its receptor on TC determining TC activation or suppression. For example, CD28:B7 co-stimulatory immune checkpoint is essential for TC expansion and differentiation [35].

Signal 3 (cytokine stimulation) mediates cytokine-induced TC expansion and differentiation. For example, IL-12 and IFN-α/β, along with Ag and immune checkpoint, enhanced CD8^+^ TC clonal expansion [36]. The combination of IL-1β and IL-6 induced T helper (Th)-17 cell differentiation from human naïve TC (CD4^+^CD45RA^+^CCR7^+^CD25^−^), in the presence of anti-CD3 (signal 1) and anti-CD28 (signal 2) antibodies [13]. IL-1β enhanced Th1, Th2, and Th17 cell proliferation with Ag stimulation in IL-1R1^−/−^Rag1^−/−^ mouse [37].

Signal 4 (MADS recognition) is a novel signal we proposed based on our and other’s recent findings [15, 26]. Metabolic RF stimulates the expression of immune checkpoint molecules via a metabolic sensor, which in turn activates APC or TC and increases inflammatory cytokine production. We reported that uremic toxin, HHcy, and S-adenosylhomocysteine (SAH) increased CD40^+^ MC and sCD40L levels during a chronic time frame of CKD patients [15]. CD40:sCD40L molecular pair further promoted pro-inflammatory CD40^+^ MC and intermediate MC differentiation in 3 days. Moreover, studies in human subjects support that signal 4 MADS recognition may be involved in TC-related adaptive immunity in metabolic disorders [38]. The levels of sCD40L were found to be increased in subjects with metabolic syndrome and hypertension and negatively related to insulin sensitivity [39]. In addition, glucose sustains TC growth and proliferation upon TCR-dependent TC activation [40].

### BC immunity (humoral immunity) (Fig. 2b)

BC immunity involves the same four signals which leads to antibody production and BC activation [41]. Signal 1 (Ag recognition) is the engagement of Ag with Ag-specific BC receptor (BCR). Signal 2 (immune checkpoint) is the ligation of immune checkpoint molecular pairs. Signal 3 (cytokine stimulation) describes Ag- and immune checkpoint-associated inflammatory cytokine regulation in BC activation. We proposed signal 4 (MADS recognition) for BC activation because the CD40:CD40L immune checkpoint is involved in BC activation [42] and sCD40L is induced in metabolic disease including CKD, HHcy, hypertension, hyperglycemia, and dyslipidemia [15, 39, 43].

## Immune checkpoint regulates TC and APC activation

**Fig. 3:**
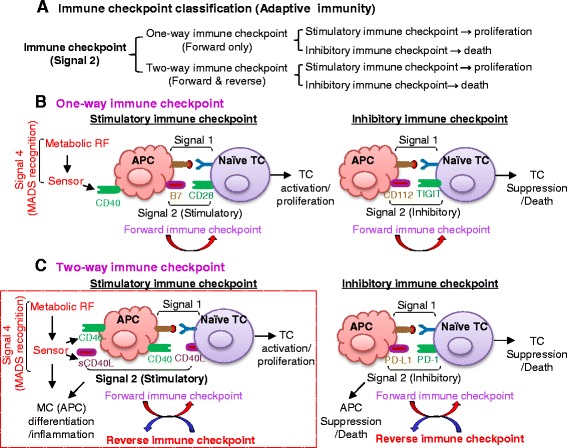
Immune checkpoint and its characterizations. **a** Immune checkpoint classification. Immune checkpoints are classified as one-way immune checkpoint and two-way immune checkpoint based on signal 2 direction and are further divided into stimulatory and inhibitory immune checkpoints. **b**. One-way immune checkpoint. The one-way immune checkpoint only involves forward signaling in signal 2. The ligation of co-stimulatory pair molecules triggers forward stimulatory immune checkpoint inducing TC proliferation, whereas the inhibitory immune checkpoint induces TC suppression or death. **c** Two-way immune checkpoint. The two-way immune checkpoint involves both forward and reverse stimulatory signaling. The reverse stimulatory immune checkpoint induces either TC proliferation or MC (APC) differentiation/inflammation. The inhibitory immune checkpoint leads to TC suppression/death or APC death. *Words in red* emphasize our newly proposed signal. Abbreviations: *APC* antigen present cell, *MC* monocyte, *RF* risk factor, *sCD40L* soluble CD40 ligand, *TC* T cell

Immune checkpoints are molecular pairs (receptor:ligand) interactions regulating immune response towards TC and APC, also termed signal 2 (Fig. 2). We classified the immune checkpoint into two categories: one-way immune checkpoint for forward signaling towards TC only, and two-way immune checkpoint for both forward and reverse signaling towards TC and APC, respectively (Fig. 3). Each category can be further divided into stimulatory and inhibitory immune checkpoints. The stimulatory immune checkpoint turns up the immune system leading to immune cell proliferation or activation, while the inhibitory immune checkpoint turns down the immune system leading to immune cell suppression or death (Fig. 3a).

### One-way immune checkpoint

One-way immune checkpoint refers to forward signaling only towards TC. It is dual-functional as it can modulate cell fate for proliferation or death (Fig. 3b).

Forward stimulatory molecular pairs promote TC proliferation, cytokine production, differentiation, cytotoxic function, memory formation, and survival. A well-described forward stimulatory molecular pair is CD28:B7. Interaction of CD28:B7 results in distinct phosphorylation, transcriptional activation, and cytokine and chemokine production that are essential for TC expansion and differentiation [35]. Metabolic product ceramide is involved in the forward stimulatory immune checkpoint in TCR-dependent TC activation at multiple levels [44].

The forward inhibitory molecular pair ligation in the one-way immune checkpoint leads to TC tolerance, exhaustion, apoptosis, cell cycle arrest, and effector function inhibition. For example, CD8^+^ tumor-infiltrating lymphocytes exhibit high proliferation and IL-2/tumor necrosis factor (TNF)-α production in TC immunoreceptor with Ig and ITIM domain (TIGIT)^−/−^ mice [45], indicating TIGIT inhibited the effector function and proliferation of CD8^+^ TC.

### Two-way immune checkpoint

The two-way immune checkpoint is bi-directional, towards both TC and APC. Similar as the one-way immune checkpoint, it is also dual-functional as it modulates cell fate for proliferation or death (Fig. 3c).

The stimulatory molecular pairs in the two-way immune checkpoint activate TC and APC. CD40:CD40L is one of the best-described stimulatory pairs in the two-way immune checkpoint. CD40 binds to its ligand CD40L, which is usually transiently expressed on TC [46] and modulates effector function and differentiation of TC. This is seen in CD40^−/−^APOE^−/−^ mice as they have lower effector memory CD4^+^/CD8^+^ TC in the spleen [47]. Ligation of CD40L on TC with CD40 on BC promoted BC Ig isotype switching, which was associated with X-linked hyper IgM syndrome in humans [48]. Moreover, metabolic RF cholesterol crystal is required for TCR nanoclustering in TC, which enhances the avidity of the TCR-antigen interaction [49]. Reversely, cholesterol crystals trigger pro-inflammatory cytokine secretion from APC MØ [50].

The inhibitory molecular pairs in the two-way immune checkpoint lead to TC and APC suppression or death. The ligation of PD-1 and PD-L1 results in TC inactivation, IL-12 reduction, antitumor immunity suppression, and tumor progression [51]. Thus, PD-1:PD-L1 immune checkpoint therapy using PD-1 antibodies (pembrolizumab and nivolumab) achieved great success in melanoma, bladder cancer, and gastric cancer therapy [9–11]. Further, PD-1 delivered inhibitory signals through B7-H1 on APC [52]. Again, metabolic RF cholesterol sulfate inhibited TCR signaling [53] as well as sterologenesis in APC fibroblasts [54].

## Immune checkpoint family and paired molecules

Representative paired immune checkpoint molecules (receptor:ligand) are summarized in Table 1 and listed according to immune checkpoint direction (one-way and two-way) and function (stimulatory and inhibitory). The classification of immune checkpoint families is determined by the checkpoint receptor component. Most immune checkpoint receptors are members of immunoglobulin superfamily (IgSF) and tumor necrosis factor receptor superfamily (TNFRSF), which can be further divided into specific subfamilies based on the primary amino acid sequence, protein structure, and function [52]. Notably, the majority of immune checkpoint ligands are expressed on multiple immune cells.

IgSF checkpoint receptor superfamily contains CD28, B7, CD226, TC (or transmembrane) immunoglobulin, mucin domain (TIM), and CD2/signaling lymphocytic activation molecule (SLAM) subfamilies, which participate in forward stimulatory and forward inhibitory immune checkpoints. For example, CD28 subfamily including CD28 and CD278 (inducible TC co-stimulator, ICOS) transduce stimulatory response. Other members in the CD28 subfamily, such as CTLA-4, PD-1, PD-1 homologue (PD-1H), and B and T lymphocyte attenuator (BTLA), transduce inhibitory response.

TNFRSF checkpoint receptor superfamily contains Type-V, Type-L, Type-s, and orphan subfamilies and recognizes TNF superfamily (TNFSF) molecules [52]. The common feature of TNFRSF:TNFSF is bi-directional (both forward and reverse immune checkpoint) [52].

The Type-L subfamily, also called conventional TNFRSF immune checkpoint receptors, has the most members in TNFRSF, but only CD40, herpes virus entry mediator (HVEM), death receptor 3 (DR3), and lymphotoxin-β receptor (LTBR) have a co-stimulatory function, while CD120a, CD120b, and CD95 have apoptosis function on TC [52, 55]. The Type-V subfamily, also called divergent, is the only family where all members have co-stimulatory function, including 4-1BB (CD137), OX40 (CD134), CD27, CD30, and glucocorticoid-induced TNFR-related protein (GITR) [55]. Among the Type-s subfamily, transmembrane activator and CAML interactor (TACI), B cell-activating factor receptor (BAFFR), and B cell maturation protein (BCMA) have the function of B cell activation, survival, and differentiation [52, 55]. The function of the orphan subfamily remains unclear, except that the receptor expressed in lymphoid tissues (RELT) has some evidences of stimulating TC proliferation [56].

We list six pairs of TNFRSF:TNFSF molecules in Table 1: CD40:CD40L, 4-1BB (CD137):4-1BBL, OX40 (CD134):OX40L, CD27:CD70, CD357 (GITR):GITRL, and CD30:CD30L, and discuss their characterizations in the following section.

## Two-way stimulatory immune checkpoint induces tissue and systemic inflammation

Emerging evidences suggested that the two-way stimulatory immune checkpoint is critical for TC activation and APC inflammation. We summarized recent studies elucidating two-way stimulatory immune checkpoint with immune cell responses in human and mouse disease models (Tables 2 and 3).

**Table 2:** Two-way stimulatory immune checkpoint induces tissue and systemic inflammation (human study)

Checkpoint (receptor:ligand)	Disease	Functional change	PMID#	Year
Forward immune checkpoint (towards TC)				
CD40:CD40L	ACS	Lesion CD40L^+^ TC↑	12732389	2003
	Bladder tumor	Peripheral T cell↓ to agonistic IgG1 chimeric α-CD40 Ab	25589626	2015
CD137:CD137L	AS	CD137↑ in lesion CD8^+^ TC	18285570	2008
	Head/neck cancer	CD4^+^/CD8^+^ TC proliferation maker Ki67↑ to CD137 agonist	27496866	2016
CD134:CD134L	ACS	CD134^+^ CD4^+^CD28^null^ TC express INF-γ/TNF-α,	22282196	2012
	Colorectal cancer	CD134^+^ CD8^+^ TC infiltration enhances prognostic	26439988	2015
CD27:CD70	NSCLC	Higher CD4/CD8 TC ratio in CD70^+^ tumor cell	25951351	2015
CD30:CD30L	Lymphoma	CD30^+^ Th2 proliferation↓ by CD30L	12855655	2003
GITR:GITRL	Liver tumor	Tumor-infiltrating Treg-suppressive capacity↓ by GITRL	26587321	2015
Reverse-immune checkpoint (towards APC)				
CD40:CD40L	CVD + CKD	CD40^+^ MC↑, MC inflammatory markers↑	27992630	2016
	Cancer	CD40L mediate TIS-TC induced inflammatory MC/MØ	25375372	2014
CD137:CD137L	AAS	CD137L^+^ CD14^+^ MC↑	24899613	2014
	MM	CD137 inhibit MM cell proliferation and induce death	20520765	2010
CD134:CD134L	ACS	OX40L^+^ MC↑	22282196	2012
	MLC	OX40L^+^ MLC-DC express higher CD80^+^/CD86^+^/HLA-DR	14984494	2004
CD27:CD70	Lymphoma	Anti-CD70 mediate MØ phagocytosis	17038522	2007
CD30:CD30L	Lymphoma	Anti-CD30 mediate MØ phagocytosis	17909075	2007
GITR:GITRL	AS	Plaque MØ activation↑	17067317	2006

Stimulatory immune checkpoint has been described in human and mouse metabolic diseases. For example, CD40:CD40L two-way stimulatory immune checkpoint was activated and associated with increased lesion CD40L^+^ TC in ACS and CD40^+^ MC in CKD in humans. CD40 KO reduced spleen effector memory CD4^+^/CD8^+^ TC and blood Ly6C^+^ MC and aorta M1 MΦ. Similar functional change was observed for immune checkpoint CD40:CD40L CD137:CD137L and OX40:OX40L. ↑ refers to higher population/expression level/activity. ↓ refers to lower population/expression level/activity

Abbreviation: *AAS* acute atherothrombotic stroke; *CAR* chimeric antigen receptor; *CKD* chronic kidney disease; *CVD* cardiovascular disease; *CA* carcinoma; *EC* endothelial cell; *GITR* glucocorticoid-induced TNFR-related protein; *GITRL* GITR ligand; *KPC* Kras^LSL-G12D/+^, Trp53^LSL-R172H/+^, Pdx1-Cre; *MC* monocyte; *MØ* macrophage; *mAb* agonist monoclonal antibodies; *MM* multiple myeloma; *MLC* myeloid leukemia cell; *NSCLC* non-small cell lung cancer; *NKC* natural killer cell; *SCID* severe combined immunodeficient; *TC* T cell; *Treg* regulatory T cell; *TG* transgene; *TIS* tumor induce Senescent; *VEGF* vascular endothelial growth factor

**Table 3:** Two-way stimulatory immune checkpoint induces tissue and systemic inflammation (mouse study)

Checkpoint (receptor:ligand)	Genotype	Disease	Functional change	PMID#	Year
Forward immune checkpoint (towards TC)					
CD40:CD40L	CD40^−/−^APOE^−/−^	AS	Spleen memory CD4^+^/CD8^+^ TC↓	20100871	2007
	Rag1^−/−^	Lung CA	Tumor Th1/Th17↑, Treg/Th2↓ to CD40 agonist	25651850	2015
CD137:CD137L	APOE^−/−^	AS	Lesion CD3^+^/CD8^+^ TC↑ to CD137 agonist	18285570	2008
	NSG	Osteosarcoma	Ameliorate CAR TC exhaustion to CD137	25939063	2015
CD134:CD134L	LDLR^−/−^	AS	Lesion CD3^+^ TC↓ to α-OX40L	24068673	2013
	C57BL/6	Mammary CA	Blood effector/memory TC↑ to α-OX40+Dribbles	27874054	2016
CD27:CD70	CD70^−/−^LDLR^−/−^	AS	Spleen Treg↓	27786334	2017
	CD27^−/−^	Solid tumor	Spleen Treg↓, tumor CD3^+^ TC infiltration↑	22628427	2012
CD30:CD30L	LDLR^−/−^	AS	Adventitial CD3^+^ TC↓ to α-CD30L	23087358	2012
	C57BL/6	Fibrosarcoma	CD30^+^ Vδ TC drives cancer progression	27384869	2016
GITR:GITRL	LDLR^−/−^GITRL^TG^	AS	Lymph node Terg/effector memory CD4^+^ TC↑	27444204	2016
Reverse-immune checkpoint (towards APC)					
CD40:CD40L	CD40^−/−^APOE^−/−^	AS	Blood Ly6C^+^ MC↓, aorta M1 MØ↓	20100871	2007
	KPC	Pancreatic CA	Tumor M1 MØ↑ to CD40 agonist	21436454	2011
CD137:CD137L	CD137^−/−^APOE^−/−^	AS	Aorta CD11b^+^ MC/MØ↓	25059229	2014
	C57BL/6	Liver CA	Tumor iNOS-positive MØ↑ to CD137 agonist	24789574	2014
CD134:CD134L	OX40L^−/−^APOE^−/−^	AS	VEGF-induced angiogenesis↓	20584752	2010
	C57BL/6	Sarcoma	Tumor M1 MØ↑ to CD134 agonist	22578109	2012
CD27:CD70	CD70^TG^ APOE^−/−^	AS	Circulating MC viability↓	20505312	2010
	SCID	Lymphoma	Delete MØ reduce survival to α-CD70	17038522	2007
CD30:CD30L	LDLR^−/−^	AS	Lesion MØ/BC no affect	23087358	2012
	SCID	Lymphoma	Delete MØ reduce survival to α-CD30	17909075	2007
GITR:GITRL	C57BL/6	Liver tumor	Tumor M1 MØ↑ to GITR agonist+sunitinib	26239999	2016

Stimulatory immune checkpoint has been described in human and mouse metabolic diseases. For example, CD40:CD40L two-way stimulatory immune checkpoint was activated and associated with increased lesion CD40L^+^ TC in ACS and CD40^+^ MC in CKD in humans. CD40 KO reduced spleen effector memory CD4^+^/CD8^+^ TC and blood Ly6C^+^ MC and aorta M1 MΦ. Similar functional change was observed for immune checkpoint CD40:CD40L CD137:CD137L and OX40:OX40L

Abbreviation: *AAS* acute atherothrombotic stroke; *CAR* chimeric antigen receptor; *CKD* chronic kidney disease; *CVD* cardiovascular disease; *CA* carcinoma; *EC* endothelial cell; *GITR* glucocorticoid-induced TNFR-related protein; *GITRL* GITR ligand; *KPC* Kras^LSL-G12D/+^, Trp53^LSL-R172H/+^, Pdx1-Cre; *MC* monocyte; *MØ* macrophage; *mAb* agonist monoclonal antibodies; *MM* multiple myeloma; *MLC* myeloid leukemia cell; *NSCLC* non-small cell lung cancer; *NKC* natural killer cell; *SCID* severe combined immunodeficient; *TC* T cell; *Treg* regulatory T cell; *TG* transgene; *TIS* tumor induce Senescent; *VEGF* vascular endothelial growth factor

### CD40:CD40L two-way immune checkpoint

CD40:CD40L is the first discovered stimulatory molecular pair of TNFRSF:TNFSF. CD40 is not only expressed on immune cells (BC, MC, MØ, DC) but also on a variety of somatic cells such as endothelial cell (EC), smooth muscle cell (SMC), fibroblast, and platelet [57]. CD40 was initially discovered as a surface receptor on BC binding to CD40L on TC causing TC polyclonal activation and BC proliferation/differentiation [46]. CD40L is the sole ligand for CD40 and is also known as CD154. CD40L has two forms, membrane-bound CD40L and sCD40L. Membrane-bound CD40L is expressed on activated TC, MC, MØ, platelet, mast cell, and EC [58]. sCD40L circulates in the blood and is mainly produced by platelets [59]. The CD40:CD40L two-way immune checkpoint promotes atherosclerosis and inhibits tumor progress and has been used as a cancer immunotherapy target [60–62]. sCD40L is significantly elevated in patients with cardiovascular disease (CVD) and CKD [15] and proposed as an independent predictor and biomarker for cardiovascular events after acute coronary syndrome and plaque vulnerability [63]. CD40:CD40L interactions stimulate the expression of inflammatory cytokines, adhesion molecules, chemokines, matrix degrading enzymes, and platelet tissue factor. CD40^−/−^ApoE^−/−^ mice exhibited 55% plaque reduction and less lipid-containing, collagen-rich, stable plaque, and improved reendothelialization [64]. Similarly, anti-CD40L antibody induced a stable lesion with lipid-poor, collagen-rich plaque in ApoE^−/−^ mice [65]. CD40-RNAi-lentivirus prevented plaque progression in ApoE^−/−^ mice [66].

#### CD40:CD40L forward immune checkpoint

The influence of CD40:CD40L forward immune checkpoint towards TC is well established. TC presents at all stages of atherosclerotic lesion. The major subset of TC in atherosclerotic plaques is Th1 CD4^+^ TC. CD40^−/−^ApoE^−/−^ mice have a lower effector memory CD4^+^/CD8^+^ TC in the spleen [47]. Anti-CD40L antibody reduced TC content in mouse atheroma [67]. Moreover, the CD40:CD40L immune checkpoint inhibited Treg activation, as CD40L^−/−^ bone marrow reconstitution in LDLR^−/−^ mice led to increased Treg [68], and agonistic CD40 antibody reduced Treg in Lewis lung cancer mouse model [69].

#### CD40:CD40L reverse immune checkpoint

Large amount of evidence described the impact of CD40:CD40L reverse stimulatory immune checkpoint towards APC. In the absence of CD40L on TC, BC only secrete IgM and cannot switch to other Igs (IgG, IgE, IgA). CD40L on TC binds to CD40 on MØ and leads to MØ activation and secretion of matrix metalloproteinase (MMP), pro-inflammatory cytokines (Il-12, TNF-α, IL-1β, IL-6, and IL-8), and platelet tissue factor. Similarly, CD40L gene mutation caused X-linked hyper IgM syndrome which is characterized by low or absent levels of IgG, IgE, and IgA but normal or elevated serum levels of IgM [48]. MC-derived DC from patients with coronary artery disease (CAD) expressed higher CD40 which was associated with smoking history, higher C-reactive protein, and lower high-density lipoprotein cholesterol (HDL-C) [70]. We reported that CD40^+^ MC was increased in patients with CVD and further elevated in patients with CVD+CKD. Anti-CD40L antibody significantly reduced MØ in mice [67]. CD40^−/−^ApoE^−/−^ mice exhibited lower pro-inflammatory Ly6C^+^ MC in blood and M1 MØ in the aorta [47]. Moreover, CD40 agonist activated antitumor MØ infiltration and resulted pancreatic cancer regression in mice [71].

### CD137 (4-1BB):CD137L (4-1BBL) two-way immune checkpoint

CD137 is mainly expressed on activated CD4^+^ TC and also on BC, MC, DC, and EC, while CD137L is constitutively expressed on APC and activated TC [72]. Soluble CD137 (sCD137) is elevated in human acute coronary syndrome (ACS) and atherothrombotic stroke [73, 74] and has been suggested as a prognostic biomarker for acute atherosclerotic disease. The CD137:CD137L immune checkpoint promotes vascular inflammation as CD137^−/−^ApoE^−/−^ and CD137^−/−^LDLR^−/−^ mice had reduced atherosclerotic lesions and inflammation [75] and anti-CD137 antibody decreased atherosclerosis lesion in ApoE^−/−^ mice [76].

#### CD137:CD137L forward immune checkpoint

The CD137:CD137L forward immune checkpoint promotes TC activation. CD137 is expressed predominantly in CD8^+^ TC and occasionally in CD4^+^ TC in human atherosclerotic lesions and associated with pro-inflammatory factor release such as TNF-α, IL-1β, and IFN-γ. CD137 agonist induced CD8^+^ TC infiltration in mouse atherosclerotic lesions and promoted the progression of atherosclerosis [76]. In peripheral blood mononuclear cells (PBMC), antibody against CD137 decreased TNF-α and IFN-γ production from CD4^+^CD28^null^ TC which expresses higher levels of CD137 compared with CD4^+^CD28^+^ TC [77]. The CD137:CD137L checkpoint also enhances tumor immunity, as CD137 agonist promoted CD4^+^ and CD8^+^ TC proliferation in patients with head and neck cancer [78].

#### CD137:CD137L reverse immune checkpoint

Recent research emphasized the role of the CD137:CD137L reverse stimulatory immune checkpoint on MC and MØ differentiation. Cross-linking of CD137L by CD137 on human PBMC induced IL-6, IL-8, IL-12, TNF-α, and IFN-γ production and inflammatory DC differentiation [79]. Circulating CD137L^+^CD14^+^ MC was increased in patients with acute ischemic atherosclerotic stroke [74]. CD137^−/−^ApoE^−/−^ mice have lower MC and MØ in the aorta [80]. Anti-CD137 monoclonal antibody induced iNOS-positive MØ differentiation in hepatoma tissue in mice [81].

### CD134(OX40):CD134L(OX40L) two-way immune checkpoint

CD134 is mainly expressed on activated CD4^+^ TC, CD8^+^ TC, and memory TC, while CD134L is expressed on mature APC, activated TC, and EC [82]. The levels of sOX40L were significantly increased in patients with ACS [83]. Anti-CD134L antibody significantly reduced atherosclerotic lesion in LDLR^−/−^ mice [84, 85].

#### CD134:CD134L forward immune checkpoint

Similar as CD137, CD134 is highly expressed in CD4^+^CD28^null^ TC. CD134 also regulates Treg function by suppressing Treg generation from naïve TC and effector TC in mice [86]. CD134L induced INF-γ CD4^+^ TC proliferation in cultured splenocytes from ApoE^−/−^ mice [87]. Antibody against CD134 decreased TNF-α and IFN-γ production in CD4^+^CD28^null^ TC derived from PBMC from ACS patients [77]. Anti-CD134L antibody reduced the populations of circulating CD4^+^CD134^+^ TC, CD4^+^ TC and CD8^+^ TC, and lesion CD3^+^ TC in LDLR^−/−^ mice [84]. Anti-CD134 antibody combined with autophagosomes (DRibbles) induced memory and effector TC proliferation and differentiation and promoted tumor regression in mice [88]. Elevating CD134^+^ CD8^+^ TC infiltration in colorectal cancer prolonged overall survival in humans [89].

#### CD134:CD134L reverse immune checkpoint

Even though circulating MC expressed the highest level of CD134L in ACS patients [77], the atherogenic role of CD134:CD134L may not be mediated by MC and MØ. Anti-CD134L antibody had no effect on both M1 MØ and M2 MØ in ApoE^−/−^ mice [87]. CD134:CD134L may participate in BC Ig isotype switch, as blocking the CD134:CD134L immune checkpoint using anti-CD134L antibody increased anti-ox-LDL IgM, a protective IgM, in LDLR^−/−^ mice [85]. Moreover, agonistic CD134 antibody increased M2 MØ in tumor. M2 MØ produced higher IL-10 and chemokine (C-C motif) ligand (CCL)-17 and lower IL-12-b and IL-23 compared to M1 MØ, which limited the efficacy of CD134 agonist therapy in mice [90, 91].

### CD27:CD70 two-way immune checkpoint

In contrast to CD134 and CD137, CD27 is expressed on naïve TC, BC, and NK cells and upregulated on activated TC, while CD70 is expressed on APC and activated TC [92]. Evidence for the role of CD27:CD70 in atherosclerosis is conflicting as ruptured atherosclerotic plaques expressed higher CD70 than those in stable lesions [93], and CD70 transgenic mice attenuated atherosclerotic development [94].

#### CD27:CD70 forward immune checkpoint

CD27 promotes activated TC proliferation and survival. CD27^+^ Treg is reduced in myocardial infarction patients, and this subset has high suppressive potential [95]. CD70 deficiency reduced spleen Treg in ApoE^−/−^ mice [93] and CD27 deficiency reduced Treg in solid tumor in mice [96], suggesting that CD27:CD70 may have an immunosuppressive role in atherosclerosis and tumor growth.

#### CD27:CD70 reverse immune checkpoint

The CD27:CD70 reverse stimulatory immune checkpoint towards APC may be protective for atherosclerosis. CD70 transgenic mice displayed increased MC apoptosis [94]. CD70 promoted ox-LDL efflux in MØ [93] while engineered anti-CD70 increased MØ phagocytosis and prolonged the survival in lymphoma mice [97].

### CD30:CD30L two-way immune checkpoint

CD30 is expressed on activated TC and BC, while CD30L is expressed on APC and activated TC [98]. CD30 was originally recognized as a cancer-associated surface antigen in TC. The CD30:CD30L two-way immune checkpoint promotes atherosclerosis and tumor and is a therapeutic target for both diseases. The CD30 antibody is used to treat Hodgkin’s lymphoma, anaplastic large cell lymphoma, and other cancers [99]. A few studies demonstrated that the CD30:CD30L blockade delayed the development of atherosclerosis.

#### CD30:CD30L forward immune checkpoint

CD30 primarily promotes CD4^+^ TC activation. Anti-CD30L treatment reduced CD4^+^ TC counts but had no effect on CD8^+^ TC, Th1, Th2, Th17, and Treg cell numbers in LDLR^−/−^ mice [100]. Recombinant CD30L inhibited CD30^+^ Th2 lymphoma cell proliferation [101].

#### CD30:CD30L reverse immune checkpoint

CD30:CD30L may not affect APC function in atherosclerosis, as anti-CD30L treatment did not change BC counts, ox-LDL-specific IgM/IgG, and aortic MC numbers in LDLR^−/−^ mice [100]. Anti-CD30 antibody enhanced MØ phagocytosis in tumor tissue and increased survival in mice [102].

### CD357(GITR):CD357L(GITRL) two-way immune checkpoint

GITR is expressed on naïve TC, increased on activated TC, and is also present on BC and NK cells, while GITR ligand (GITRL) is expressed on APC [103]. GITR:GITRL may have a protective role in atherosclerosis via regulating Treg. GITRL transgenic BM transplantation reduced atherosclerotic lesion in LDLR^−/−^ mice [104].

#### GITR:GITRL forward immune checkpoint

It is known that GITR:GITRL interaction is important for CD4^+^ TC, CD8^+^ TC, and Treg differentiation and expansion. Thus, GIRT is used as a Treg marker. GITRL transgenic chimeric LDLR^−/−^ mice displayed an increased effector TC and Treg and reduced atherosclerosis [104]. sGITRL suppressed Treg infiltration in human liver tumor [105].

#### GITR:GITRL reverse immune checkpoint

GITR and GITRL are mainly expressed in MØ in plaques. However, the protective role of GITR:GITRL in atherosclerosis is controversial. Anti-GITR mAb induced human MC and MØ activation, MMP-9, and pro-inflammatory cytokine expression, which may promote atherosclerosis and plaque instability [106]. Agonistic GITR antibody promoted M1 MØ differentiation in mice liver tumor [107].

## Molecular mechanisms underlying CD40:CD40L two-way immune checkpoint amplification

**Fig. 4:**
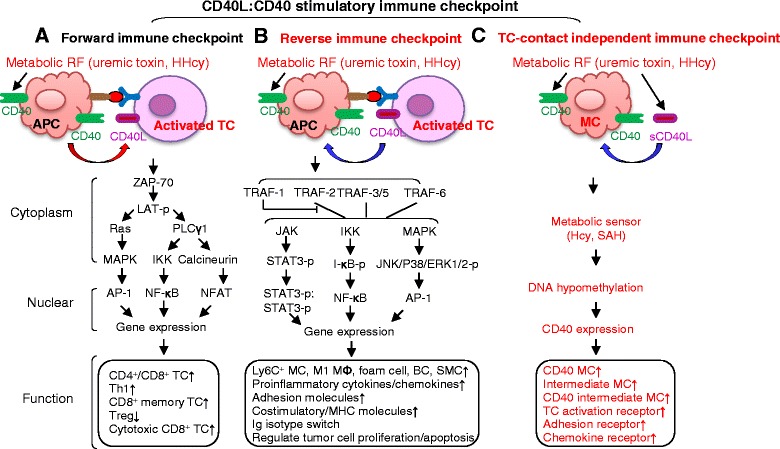
CD40:CD40L stimulatory immune checkpoint (molecular mechanism and biological function). **a** Forward immune checkpoint. CD40:CD40L stimulation occurs when B7 engages CD28. In TC, CD40:CD40L ligation, via ZAP-70 activation, leads to activating three important signal pathways (MAPK/NF-κB/calcineurin) and promotes gene transactivation and TC activation. **b** Reverse-immune checkpoint. In APC, CD40:CD40L ligation, via TRAF2/3/5/6 activation and the following STATS, NF-κB, and AP-1 activation, promotes gene expression and APC inflammation. **c** TC contact-independent immune checkpoint. Metabolic RF increases circulating sCD40L and CD40 in MC. sCD40L:CD40 co-stimulation results in CD40 MC differentiation and inflammation via metabolic sensor and DNA hypomethylation-related mechanisms. *Words in red* emphasize our new findings and proposed signal. Abbreviations: *APC* antigen present cell, *AP-1* activator protein 1, *BC* B cell, *ERK* extracellular signal-regulated kinase, *HHcy* hyperhomocysteinemia, *Ig* immunoglobulin, *IKK* I-κB kinase, *I-κBs* I-κB proteins, *JNK* JUN amino-terminal kinase, *LAT* linker for activation of T cells, *MAPK* mitogen-activated protein kinase, *MØ* macrophage, *MC* monocyte, *NKC* natural killer cell, *NF-κB* nuclear factor κB, *p* phosphorylated, *PLCγ1* phospholipase C gamma 1, *sCD40L* soluble CD40 ligand, *SMC* smooth muscle cell, *STAT3* signal transducers and activator of transcription-3, *TC* T cell, *Treg* regulatory T cell, *TRAF* tumor necrosis factor receptor, *ZAP70* zeta chain-associated protein kinase

We summarized two molecular signaling pathways previously reported for the CD40:CD40L immune checkpoint: forward stimulatory immune checkpoint towards TC and reverse stimulatory immune checkpoint towards APC (Fig. 4a, b). In addition, we propose a novel pathway, the TC contact-independent immune checkpoint (Fig. 4c) based on our recent discoveries [15].

We found that metabolic RF, such as uremic toxin and HHcy, induced circulating sCD40L and CD40^+^ MC in CKD patients. Also, both sCD40L and HHcy promoted inflammatory CD40^+^ MC and intermediate MC differentiation in cultured human PBMC [15]. Other metabolic RF, such as triazolopyrimidine, inhibited CD40-associated MC activation [108]. A mechanistic study showed that SAH-related DNA hypomethylation is responsible for CD40^+^ MC differentiation in human PBMC [15]. We were the first to establish a direct mechanistic link between HHcy and increased cellular SAH and to propose that SAH-related hypomethylation is a key biochemical mechanism for HHcy-induced CVD in EC [109–111]. We believe that Hcy and SAH function as metabolic sensors and are responsible for DNA hypomethylation and APC activation.

### CD40:CD40L forward stimulatory immune checkpoint (Fig. 4a)

The CD40:CD40L forward stimulatory immune checkpoint follows signal 1 (Ag recognition) and leads to TC activation. During this process, MHC presents Ag to TCR, which triggers the assembling of TCR, CD3, and TCRζ chain. The subsequent CD40:CD40L immune checkpoint interaction amplifies the activation of three transduction pathways via the recruitment of zeta chain-associated protein kinase of 70 kDa (ZAP-70) and phosphorylation of linker for activation of T cells (LAT), RAS mitogen-activated protein kinase (MAPK) pathway, calcium-calcineurin pathway, and nuclear factor κB (NF-κB) pathway [112].

### CD40:CD40L reverse stimulatory immune checkpoint (Fig. 4b)

In APC, the CD40:CD40L reverse stimulatory immune checkpoint is associated with proliferation of MC, MØ, BC, SMC, and tumor cells, and inflammatory molecular production. CD40 can bind to TNF receptor-associated factor (TRAF1-3/5-6) and activate three TNF signaling, including signal transducers and activator of transcription-3 (STAT3), NF-κB, and activator protein 1 (AP-1) pathways in cell type and TRAF member-dependent manner. For example, STAT3 can be activated by CD40:TRAF2/3 ligation via JAK in BC [113]; NF-κB can be activated by CD40:TRAF1-3/5-6 interaction via IKK/I-κB in BC and MC; and AP-1 can be activated by CD40:TRAF6 via MAPK in MC and MØ [114]. Moreover, TRAF1/2/3/5 activation is linked to NF-κB, MAPK/p38, and JUN amino-terminal kinase (JNK) pathway, while TRAF6 activates NF-κB, protein kinase B, and STAT3 pathway [113]. CD40:TRAF6 has a critical role in promoting atherosclerosis, as attenuated atherosclerosis and reduced Ly6C^+^ MC and M1 MØ were observed in CD40^−/−^TRAF6^−/−^ApoE^−/−^ but not in CD40^−/−^TRAF2/3/5^−/−^ApoE^−/−^ mice [47].

### CD40:CD40L TC contact-independent immune checkpoint (Fig. 4c)

This is a novel pathway we proposed based on our and others discoveries [15, 115]. We demonstrated that metabolic RF, such as uremic toxin and HHcy, and sCD40L promoted inflammatory CD40^+^ MC and intermediate MC differentiation in culture human PBMC in the absence of TC [15]. We hypothesize that metabolic RF promote pro-inflammatory MC differentiation via metabolic sensors, such Hcy and SAH and DNA hypomethylation. This is based on evidence from mediation analysis showing increased plasma Hcy and SAH levels and consequential reduction of SAM/SAH ratio, a recognized indicator of methylation status, and from mechanistic studies showing Hcy-suppressed DNA methylation in CD40 promoter and folic acid, a methylation rescue reagent, reversed CD40^+^ MC differentiation in human PBMC [15]. We were the first to establish a direct mechanistic link between Hcy and increased cellular SAH with hypomethylation and to propose hypomethylation as a key biochemical mechanism for HHcy-induced CVD [109–111]. Our discoveries suggested that the TC contact-independent immune checkpoint is a critical mechanism for systemic and tissue inflammatory response in metabolic disorders.

## CD40^+^ MC is a novel and stronger inflammatory MC subset

MC heterogeneity has been widely acknowledged. MC expresses various receptors, which sense the environment stimulation and mediate cell differentiation towards inflammatory or anti-inflammatory subsets. MC is the most invasive immune cells which can transmigrate into tissue causing tissue inflammation and repair. In humans, MC are divided into three functionally distinct subsets according to the surface marker CD14 and CD16 [116]. CD14 is used as a marker for human MC. The common MC subsets by nomenclature is classified as (1) classical MC (CD14^++^CD16^−^ phagocytic MC), (2) intermediate MC (CD14^++^CD16^+^ pro-inflammatory MC), and (3) non-classical MC (CD14^+^CD16^++^ patrolling MC) [116]. However, such human MC classification is not in harmony, as further increased expression of CD16, an inflammatory marker, is associated with anti-inflammatory function in non-classical MC subsets.

Our recent findings resolved the above controversy in MC subset classification and presented CD40^+^ MC as a novel and stronger pro-inflammatory MC subset compared with the nomenclature-defined intermediate MC (Table 4) [15]. By examining the expression of nine inflammatory markers in three nomenclature-defined MC subsets and CD40^+^ MC [15], we discovered that CD40^+^ MC expressed higher levels of TC activation receptor CD86, chemokine receptor CCR2, and expressed similar levers of other inflammatory surface markers than that on nomenclature-defined intermediate MC (Table 4). In contrast, CD40^−^ MC exhibited much lower levels of TC activation receptor HLA-DR, adhesion receptor CD49d, and chemokine receptor CX3CR1 than that on common nomenclature-recognized anti-inflammatory (patrolling) non-classical MC subset.

**Table 4:** CD40^+^ MC is a novel and stronger pro-inflammatory MC subset compared with intermediated MC

Inflammatory surface maker	Pro-inflammatory MC		Anti-inflammatory MC	
	CD40^+^ MC (CD40^+^CD14^+^)	Intermediate MC (CD14^++^CD16^+^)	CD40^−^ MC (CD40^−^CD14^+^)	Non-classical MC (CD14^+^CD16^++^)
TC activation receptor				
CD86	+++	++	+	+
CD80	+	+	+	+
LA-DR	++	++	+	+++
Adhesion receptor				
CD62L	++	++	++	+
CD11b	+++	+++	+	+
CD49d	+++	+++	+	+++
Chemokine receptor				
CCR2	+++	+	++	+
CCR5	+++	+++	+	+
CX3CR1	+++	+++	++	+++

Inflammatory features of human CD40^+^ MC were characterized using nine inflammatory surface makers by flow cytometry analysis (experimental details in Yang et al. [15]). WBC from healthy subjects were isolated and stained with anti-CD14, anti-CD16, and anti-CD40 antibodies and co-stained with surface markers for TC activation (CD86, CD80, and HLA-DR), adhesion receptors (CD62L, CD11b, and CD49d), and chemokine receptors (CCR2, CCR5, and CX3CR1). Compared with the previously established inflammatory intermediate MC subset, CD40^+^ MC expressed higher levels of CD86 and CCR2 and similar levels of other inflammatory markers. CD40^−^ MC expressed much lower levels of HLA-DR, CD49d, and CX3CR1 and similar levels of other inflammatory markers compared with non-classic MC

Abbreviations: *MC* monocyte, *TC* T cell, *WBC* white blood cells

On the other hand, classically defined pro-inflammatory intermediate MC expressed lower levels of inflammatory markers CCR2, HLA-DR, and CD62L compared with classical (phagocytic) and non-classical (patrolling) MC [15]. This is inconsistent with the inflammatory feature of these MC subsets.

## CD40^+^ MC is a reliable biomarker of CKD severity

CKD is considered as a metabolic complication. Patients with CKD have 10 to 30 times higher cardiovascular mortality than the general population, and 50% of deaths in end-stage CKD were due to CVD [117]. MC is the key player in the development of atherosclerosis. Intermediate MC was elevated in patients with CVD compared with healthy subjects [15] and in patients with ST-elevation myocardial infarction. Its population is positively correlated with cardiovascular events, such as cardiovascular death, acute myocardial infarction, and non-hemorrhagic stroke [118]. However, there are a few contradictory dilemmas regarding the molecular marker and biological function of the currently defined three MC subsets [15]. For example, (1) the intermediate CD14^++^CD16^+^ (pro-inflammatory) MC expresses very high levels of anti-inflammatory marker CX3CR1 and (2) high levels and absence of CD16 were presented in CD14^+^CD16^++^ (phagocytic) and CD14^++^CD16^−^ (patrolling) MC, respectively, both displaying an anti-inflammatory function. Therefore, there is no strong consensus to use the intermediate CD14^++^CD16^+^ MC as a reliable biomarker for the severity of CVD and metabolic disease.

**Fig. 5:**
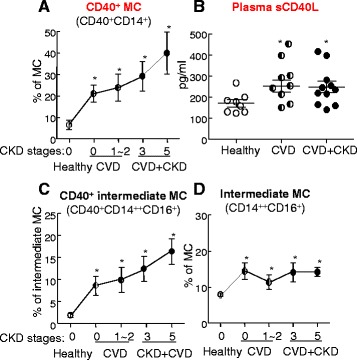
CD40^+^ MC is a reliable biomarker for CKD severity. CD40^+^ MC and plasma sCD40L were examined in human CKD subjects (experimental details in Yang et al. [15]). Peripheral WBC were isolated after red blood cell lysis and stained with antibodies against CD14, CD16, and CD40 for flow cytometry analysis. **a** CD40^+^ MC. CD40^+^ MC subset was elevated in patients with CVD and CVD+CKD compared to healthy subjects and increased with CKD severity. **b** Plasma sCD40L. sCD40L was elevated in patients with CVD and CVD+CKD compared to healthy subjects. **c** CD40^+^ intermediate MC. CD40^+^ intermediated MC subset was elevated in patients with CVD and CVD+CKD compared to healthy subjects and increased with CKD severity. **d** Intermediate MC. Intermediate MC subset was elevated in CVD patients, but not further increased in CVD+CKD patients. *p < 0.05 vs healthy. Abbreviations: *CKD* chronic kidney disease, *CVD* cardiovascular disease, *MC* monocyte, *PBMC* peripheral blood mononuclear cells, *sCD40L* soluble CD40 ligand, *WBC* white blood cells

Currently, CKD severity is determined by estimated glomerular filtration rate (eGFR) which is a prediction parameter calculated using blood creatinine, age, race, gender, and other factors. We believe CD40^+^ MC is a more accurate and reliable biomarker for CKD and CVD [15]. As shown in Fig. 5, CD40^+^ MC subset was elevated in patients with CVD and CVD+CKD compared with healthy subjects and increased with the elevation of CKD severity (Fig. 5a). Similarly, sCD40L was also elevated in patients with CVD and CVD+CKD compared to healthy subjects (Fig. 5b). CD40^+^ intermediated MC subset was elevated in patients with CVD and CVD+CKD compared with healthy subjects and increased with the elevation of CKD severity (Fig. 5c). However, intermediate MC subset was elevated in CVD patients but not further increased in CVD+CKD patients (Fig. 5d). Future studies will further analyze the relationship of CD40^+^ MC with different subtypes of CKD; such studies should allow us to better define CD40^+^ MC as a diagnosis and prognosis biomarker for CKD.

## Conclusion

**Fig. 6:**
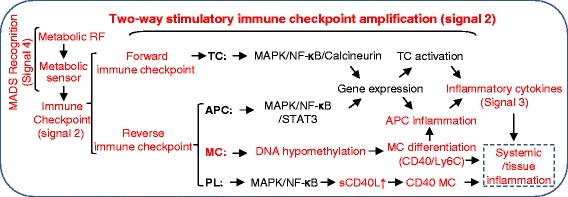
Working model of metabolic risk factor-induced two-way stimulatory immune checkpoint amplification and systemic/tissue inflammation. Metabolic risk factors, such as HHcy, uremic toxins, and other RF,, stimulate two-way stimulatory immune checkpoint amplification in TC, APC (MC), and possibly in PL via MADS recognition. In response to metabolic RF stimulation, metabolic sensors mediate TC activation via MAPK/NF-κB/calcineurin pathway, APC inflammation via STAT3MAPK/NF-κB pathway, MC differentiation via DNA hypomethylation, and possibly sCD40L production in PL via MAPK/NF-κB activation. TC activation and APC inflammation finally result in inflammatory cytokine production and systemic/tissue inflammation. *Words in red* emphasize our newly proposed signal pathway. Abbreviation: *APC* antigen present cell, *HHcy* hyperhomocysteinemia, *MC* monocyte, *MAPK* mitogen-activated protein kinase, *MADS* metabolism-associated danger signal, *NF-κB* nuclear factor κB, *RF* risk factor, *PL* platelet, *STAT3* signal transducers and activator of transcription-3, *sCD40L* soluble CD40 ligand, *TC* T cell

A novel metabolic response was incorporated into the immune system framework, providing an extensive overview of current knowledge in immune checkpoint theory (Fig. 6). This metabolic response is a novel MADS recognition pattern, mediating metabolic RF-induced innate and adaptive immune response. We propose the MADS recognition as signal 4 in adaptive immunity. MADS recognition induces immune checkpoint molecule expression via metabolic sensor leading to amplification of signal 2 two-way stimulatory immune checkpoint amplification. The forward immune checkpoint leads to TC activation. The reverse immune checkpoint leads to APC activation. Metabolic RF, such as uremic toxin or HHcy, was demonstrated to induce CD40 expression in MC and to elevate circulating sCD40L resulting in CD40^+^ MC differentiation via metabolic sensor. We defined CD40^+^ MC as a novel and stronger pro-inflammatory MC subset, compared with intermediate MC, and a reliable biomarker for CKD severity. Our studies supported the notion that MADS recognition amplify stimulatory immune checkpoint leading to TC activation and APC inflammation, respectively, which results in systemic and tissue inflammation. Furthermore, we propose the CD40:CD40L immune checkpoint as a therapeutic target for metabolic disease, CVD, and cancer.

## Additional file

Additional file 1: Table S1. Features of innate and adaptive immunity. (DOC 29 kb)

## Abbreviations

Ab: Antibody
Ag: Antigen
AP-1: Activator protein 1
APC: Antigen-presenting cell
BC: B cell
BCR: B cell receptor
BTLA: B and T lymphocyte attenuator
CKD: Chronic kidney disease
CpG: C, a cytosine triphosphate deoxynucleotide
CRTAM: Cytotoxic and regulatory T cell molecule
CTL: Cytotoxic T lymphocytes
CTLA-4: Cytotoxic T lymphocyte-associated protein 4
CVD: Cardiovascular disease
DAMP: Danger-associated molecular patterns
DC: Dendritic cell
DNAM-1: DNAX accessory molecule-1
EC: Endothelial cell
EPC: Epithelial cell
ERK: Extracellular signal-regulated kinase
FIB: Fibroblast
Foxp3: Forkhead box P3
GITR: Glucocorticoid-induced TNFR-related protein
GITRL: GITR ligand
HHcy: Hyperhomocysteinemia
HVEM: Herpes virus entry mediator
ICOS: Inducible T cell co-stimulator
IFN: Interferon
Ig: Immunoglobulin
IKK: I-κB kinase
IL: Interleukin
I-κBs: I-κB proteins
JNK: JUN amino-terminal kinase
LAT: Linker for activation of T cells
LPS: Lipopolysaccharide
MADS: Metabolism-associated danger signal
MAPK: Mitogen-activated protein kinase
MC: Monocyte
MHC: Major histocompatibility complex
MØ: Macrophage
NECL2: Nectin-like protein 2
NF-κB: Nuclear factor κB
NK: Natural killer
NLR: Nucleotide-binding and oligomerization domain-like receptors
Pam_3_CSK_4_: Tripalmitoyl-S-glycero-Cys-(Lys)4
PAMP: Pathogen-associated molecular patterns
PBMC: Peripheral blood mononuclear cells
PD-1: Programmed cell death protein 1
PD-L: PD ligand
PLCγ1: Phospholipase C gamma 1
Poly(I:C): Polyinosinic-polycytidylic acid
PRR: Pattern recognition receptor
RF: Risk factors
sCD40L: Soluble CD40 ligand
STAT3: Signal transducers and activator of transcription-3
TC: T cell
TCR: T cell receptor
TGF-β: Transforming growth factor beta
Th: T helper cell
TIGIT: T cell immunoreceptor with Ig and ITIM domains
TIM: T cell (or transmembrane) immunoglobulin and mucin domain
TLR: Toll-like receptors
TNF: Tumor necrosis factor
TNFSF: Tumor necrosis factor superfamily
TRAF: Tumor necrosis factor receptor
Treg: Regulatory T cell
VEGF: Vascular endothelial growth factor
WBC: White blood cells
ZAP70: Zeta chain-associated protein kinase

## References

[1] Yang XF, Yin Y, Wang H (2008). Vascular inflammation and atherogenesis are activated via receptors for PAMPs and suppressed by regulatory T cells. Drug Discov Today Ther Strateg.

[2] Peng G, Guo Z, Kiniwa Y, Voo KS, Peng W, Fu T, Wang DY, Li Y, Wang HY, Wang RF (2005). Toll-like receptor 8-mediated reversal of CD4+ regulatory T cell function. Science.

[3] Gerriets VA, Kishton RJ, Johnson MO, Cohen S, Siska PJ, Nichols AG, Warmoes MO, de Cubas AA, MacIver NJ, Locasale JW (2016). Foxp3 and Toll-like receptor signaling balance Treg cell anabolic metabolism for suppression. Nat Immunol.

[4] Hansson GK, Libby P, Schonbeck U, Yan ZQ (2002). Innate and adaptive immunity in the pathogenesis of atherosclerosis. Circ Res.

[5] Jenkins MK, Taylor PS, Norton SD, Urdahl KB (1991). CD28 delivers a costimulatory signal involved in antigen-specific IL-2 production by human T cells. J Immunol.

[6] Nakanishi K, Yoshimoto T, Tsutsui H, Okamura H (2001). Interleukin-18 is a unique cytokine that stimulates both Th1 and Th2 responses depending on its cytokine milieu. Cytokine Growth Factor Rev.

[7] Brahmer JR, Tykodi SS, Chow LQ, Hwu WJ, Topalian SL, Hwu P, Drake CG, Camacho LH, Kauh J, Odunsi K (2012). Safety and activity of anti-PD-L1 antibody in patients with advanced cancer. N Engl J Med.

[8] Ford ML, Larsen CP (2009). Translating costimulation blockade to the clinic: lessons learned from three pathways. Immunol Rev.

[9] Ma W, Gilligan BM, Yuan J, Li T (2016). Current status and perspectives in translational biomarker research for PD-1/PD-L1 immune checkpoint blockade therapy. J Hematol Oncol.

[10] Tsai KK, Daud AI (2015). Nivolumab plus ipilimumab in the treatment of advanced melanoma. J Hematol Oncol.

[11] Wang J, Yuan R, Song W, Sun J, Liu D, Li Z (2017). PD-1, PD-L1 (B7-H1) and tumor-site immune modulation therapy: the historical perspective. J Hematol Oncol.

[12] Ceeraz S, Nowak EC, Burns CM, Noelle RJ (2014). Immune checkpoint receptors in regulating immune reactivity in rheumatic disease. Arthritis Res. Ther..

[13] Acosta-Rodriguez EV, Napolitani G, Lanzavecchia A, Sallusto F (2007). Interleukins 1beta and 6 but not transforming growth factor-beta are essential for the differentiation of interleukin 17-producing human T helper cells. Nat Immunol.

[14] Topalian SL, Drake CG, Pardoll DM (2015). Immune checkpoint blockade: a common denominator approach to cancer therapy. Cancer Cell.

[15] Yang J, Fang P, Yu D, Zhang L, Zhang D, Jiang X, Yang WY, Bottiglieri T, Kunapuli SP, Yu J (2016). Chronic kidney disease induces inflammatory CD40+ monocyte differentiation via homocysteine elevation and DNA hypomethylation. Circ Res.

[16] Xi H, Zhang Y, Xu Y, Yang WY, Jiang X, Sha X, Cheng X, Wang J, Qin X, Yu J (2016). Caspase-1 inflammasome activation mediates homocysteine-induced pyrop-apoptosis in endothelial cells. Circ Res.

[17] Zhang D, Jiang X, Fang P, Yan Y, Song J, Gupta S, Schafer AI, Durante W, Kruger WD, Yang X (2009). Hyperhomocysteinemia promotes inflammatory monocyte generation and accelerates atherosclerosis in transgenic cystathionine beta-synthase-deficient mice. Circulation.

[18] Zhang D, Fang P, Jiang X, Nelson J, Moore JK, Kruger WD, Berretta RM, Houser SR, Yang X, Wang H (2012). Severe hyperhomocysteinemia promotes bone marrow-derived and resident inflammatory monocyte differentiation and atherosclerosis in LDLr/CBS-deficient mice. Circ Res.

[19] Fang P, Zhang D, Cheng Z, Yan C, Jiang X, Kruger WD, Meng S, Arning E, Bottiglieri T, Choi ET (2014). Hyperhomocysteinemia potentiates hyperglycemia-induced inflammatory monocyte differentiation and atherosclerosis. Diabetes.

[20] Mai J, Virtue A, Shen J, Wang H, Yang XF (2013). An evolving new paradigm: endothelial cells–conditional innate immune cells. J Hematol Oncol.

[21] Li YF, Ren LN, Guo G, Cannella LA, Chernaya V, Samuel S, Liu SX, Wang H, Yang XF (2015). Endothelial progenitor cells in ischemic stroke: an exploration from hypothesis to therapy. J Hematol Oncol.

[22] Wang X, Li YF, Nanayakkara G, Shao Y, Liang B, Cole L, Yang WY, Li X, Cueto R, Yu J (2016). Lysophospholipid receptors, as novel conditional danger receptors and homeostatic receptors modulate inflammation-novel paradigm and therapeutic potential. J Cardiovasc Transl Res.

[23] Liu W, Yin Y, Zhou Z, He M, Dai Y (2014). OxLDL-induced IL-1 beta secretion promoting foam cells formation was mainly via CD36 mediated ROS production leading to NLRP3 inflammasome activation. Inflammation research: official journal of the European Histamine Research Society [et al].

[24] Wang L, Fu H, Nanayakkara G, Li Y, Shao Y, Johnson C, Cheng J, Yang WY, Yang F, Lavallee M (2016). Novel extracellular and nuclear caspase-1 and inflammasomes propagate inflammation and regulate gene expression: a comprehensive database mining study. J Hematol Oncol.

[25] Li X, Wang S, Zhu R, Li H, Han Q, Zhao RC (2016). Lung tumor exosomes induce a pro-inflammatory phenotype in mesenchymal stem cells via NFkappaB-TLR signaling pathway. J Hematol Oncol.

[26] Junger WG (2011). Immune cell regulation by autocrine purinergic signalling. Nat Rev Immunol.

[27] Sutton CE, Lalor SJ, Sweeney CM, Brereton CF, Lavelle EC, Mills KH (2009). Interleukin-1 and IL-23 induce innate IL-17 production from gammadelta T cells, amplifying Th17 responses and autoimmunity. Immunity.

[28] Goplen NP, Saxena V, Knudson KM, Schrum AG, Gil D, Daniels MA, Zamoyska R, Teixeiro E (2016). IL-12 signals through the TCR to support CD8 innate immune responses. J Immunol.

[29] Kim HK, Falugi F, Missiakas DM, Schneewind O (2016). Peptidoglycan-linked protein A promotes T cell-dependent antibody expansion during Staphylococcus aureus infection. Proc Natl Acad Sci U S A.

[30] Secatto A, Rodrigues LC, Serezani CH, Ramos SG, Dias-Baruffi M, Faccioli LH, Medeiros AI (2012). 5-Lipoxygenase deficiency impairs innate and adaptive immune responses during fungal infection. PLoS One.

[31] Kolumam GA, Thomas S, Thompson LJ, Sprent J, Murali-Krishna K (2005). Type I interferons act directly on CD8 T cells to allow clonal expansion and memory formation in response to viral infection. J Exp Med.

[32] Flemming A (2017). T cells: successful checkpoint blockade requires positive co-stimulation. Nat Rev Immunol.

[33] Topalian SL, Weiner GJ, Pardoll DM (2011). Cancer immunotherapy comes of age. J. Clin. Oncol..

[34] Brahmer JR, Drake CG, Wollner I, Powderly JD, Picus J, Sharfman WH, Stankevich E, Pons A, Salay TM, McMiller TL (2010). Phase I study of single-agent anti-programmed death-1 (MDX-1106) in refractory solid tumors: safety, clinical activity, pharmacodynamics, and immunologic correlates. J. Clin. Oncol..

[35] Esensten JH, Helou YA, Chopra G, Weiss A, Bluestone JA (2016). CD28 costimulation: from mechanism to therapy. Immunity.

[36] Curtsinger JM, Mescher MF (2010). Inflammatory cytokines as a third signal for T cell activation. Curr Opin Immunol.

[37] Ben-Sasson SZ, Hu-Li J, Quiel J, Cauchetaux S, Ratner M, Shapira I, Dinarello CA, Paul WE (2009). IL-1 acts directly on CD4 T cells to enhance their antigen-driven expansion and differentiation. Proc Natl Acad Sci U S A.

[38] Mauro C, Marelli-Berg FM (2012). T cell immunity and cardiovascular metabolic disorders: does metabolism fuel inflammation?. Front Immunol.

[39] Gateva A, Assyov Y, Tsakova A, Kamenov Z (2016). Soluble CD40L is associated with insulin resistance, but not with glucose tolerance in obese nondiabetic patients. Arch Physiol Biochem.

[40] Palmer CS, Ostrowski M, Balderson B, Christian N, Crowe SM (2015). Glucose metabolism regulates T cell activation, differentiation, and functions. Front Immunol.

[41] Gauld SB, Merrell KT, Cambier JC (2006). Silencing of autoreactive B cells by anergy: a fresh perspective. Curr Opin Immunol.

[42] Clark EA (2014). A short history of the B-cell-associated surface molecule CD40. Front Immunol.

[43] Schonbeck U, Gerdes N, Varo N, Reynolds RS, Horton DB, Bavendiek U, Robbie L, Ganz P, Kinlay S, Libby P (2002). Oxidized low-density lipoprotein augments and 3-hydroxy-3-methylglutaryl coenzyme A reductase inhibitors limit CD40 and CD40L expression in human vascular cells. Circulation.

[44] Adam D, Heinrich M, Kabelitz D, Schutze S (2002). Ceramide: does it matter for T cells?. Trends Immunol.

[45] Kurtulus S, Sakuishi K, Ngiow SF, Joller N, Tan DJ, Teng MW, Smyth MJ, Kuchroo VK, Anderson AC (2015). TIGIT predominantly regulates the immune response via regulatory T cells. J Clin Invest.

[46] Elgueta R, Benson MJ, de Vries VC, Wasiuk A, Guo Y, Noelle RJ (2009). Molecular mechanism and function of CD40/CD40L engagement in the immune system. Immunol Rev.

[47] Lutgens E, Lievens D, Beckers L, Wijnands E, Soehnlein O, Zernecke A, Seijkens T, Engel D, Cleutjens J, Keller AM (2010). Deficient CD40-TRAF6 signaling in leukocytes prevents atherosclerosis by skewing the immune response toward an antiinflammatory profile. J Exp Med.

[48] Hill A, Chapel H (1993). X-linked immunodeficiency. The fruits of cooperation. Nature.

[49] Molnar E, Swamy M, Holzer M, Beck-Garcia K, Worch R, Thiele C, Guigas G, Boye K, Luescher IF, Schwille P (2012). Cholesterol and sphingomyelin drive ligand-independent T-cell antigen receptor nanoclustering. J Biol Chem.

[50] Grebe A, Latz E (2013). Cholesterol crystals and inflammation. Curr Rheumatol Rep.

[51] Ostrand-Rosenberg S, Horn LA, Haile ST (2014). The programmed death-1 immune-suppressive pathway: barrier to antitumor immunity. J Immunol.

[52] Chen L, Flies DB (2013). Molecular mechanisms of T cell co-stimulation and co-inhibition. Nat Rev Immunol.

[53] Wang F, Beck-Garcia K, Zorzin C, Schamel WW, Davis MM (2016). Inhibition of T cell receptor signaling by cholesterol sulfate, a naturally occurring derivative of membrane cholesterol. Nat Immunol.

[54] Williams ML, Rutherford SL, Feingold KR (1987). Effects of cholesterol sulfate on lipid metabolism in cultured human keratinocytes and fibroblasts. J Lipid Res.

[55] Croft M, Duan W, Choi H, Eun SY, Madireddi S, Mehta A (2012). TNF superfamily in inflammatory disease: translating basic insights. Trends Immunol.

[56] Sica GL, Zhu G, Tamada K, Liu D, Ni J, Chen L (2001). RELT, a new member of the tumor necrosis factor receptor superfamily, is selectively expressed in hematopoietic tissues and activates transcription factor NF-kappaB. Blood.

[57] Jansen MF, Hollander MR, van Royen N, Horrevoets AJ, Lutgens E (2016). CD40 in coronary artery disease: a matter of macrophages?. Basic Res Cardiol.

[58] Rizvi M, Pathak D, Freedman JE, Chakrabarti S (2008). CD40-CD40 ligand interactions in oxidative stress, inflammation and vascular disease. Trends Mol Med.

[59] Andre P, Nannizzi-Alaimo L, Prasad SK, Phillips DR (2002). Platelet-derived CD40L: the switch-hitting player of cardiovascular disease. Circulation.

[60] Schiza A, Wenthe J, Mangsbo S, Eriksson E, Nilsson A, Totterman TH, Loskog A, Ullenhag G (2017). Adenovirus-mediated CD40L gene transfer increases Teffector/Tregulatory cell ratio and upregulates death receptors in metastatic melanoma patients. J Transl Med.

[61] Ngiow SF, Young A, Blake SJ, Hill GR, Yagita H, Teng MW, Korman AJ, Smyth MJ (2016). Agonistic CD40 mAb-driven IL12 reverses resistance to anti-PD1 in a T-cell-rich tumor. Cancer Res.

[62] Vonderheide RH, Glennie MJ (2013). Agonistic CD40 antibodies and cancer therapy. Clin. Cancer Res..

[63] Helseth R, Weiss TW, Opstad TB, Siegbahn A, Solheim S, Freynhofer MK, Huber K, Arnesen H, Seljeflot S (2015). Associations between circulating proteins and corresponding genes expressed in coronary thrombi in patients with acute myocardial infarction. Thromb Res.

[64] Lutgens E, Gorelik L, Daemen MJ, de Muinck ED, Grewal IS, Koteliansky VE, Flavell RA (1999). Requirement for CD154 in the progression of atherosclerosis. Nat Med.

[65] Lutgens E, Cleutjens KB, Heeneman S, Koteliansky VE, Burkly LC, Daemen MJ (2000). Both early and delayed anti-CD40L antibody treatment induces a stable plaque phenotype. Proc Natl Acad Sci U S A.

[66] Wang B, Qian H, Yang H, Xu L, Xu W, Yan J (2013). Regression of atherosclerosis plaques in apolipoprotein E-/- mice after lentivirus-mediated RNA interference of CD40. Int J Cardiol.

[67] Mach F, Schonbeck U, Sukhova GK, Atkinson E, Libby P (1998). Reduction of atherosclerosis in mice by inhibition of CD40 signalling. Nature.

[68] Slawek A, Maj T, Chelmonska-Soyta A (2013). CD40, CD80, and CD86 costimulatory molecules are differentially expressed on murine splenic antigen-presenting cells during the pre-implantation period of pregnancy, and they modulate regulatory T-cell abundance, peripheral cytokine response, and pregnancy outcome. Am J Reprod Immunol.

[69] Zhang Y, Hu X, Hu Y, Teng K, Zhang K, Zheng Y, Hong X, Yu K, Wang Y, Liu L (2015). Anti-CD40-induced inflammatory E-cadherin+ dendritic cells enhance T cell responses and antitumour immunity in murine Lewis lung carcinoma. J Exp Clin Cancer Res.

[70] Dopheide JF, Sester U, Schlitt A, Horstick G, Rupprecht HJ, Munzel T, Blankenberg S (2007). Monocyte-derived dendritic cells of patients with coronary artery disease show an increased expression of costimulatory molecules CD40, CD80 and CD86 in vitro. Coron Artery Dis.

[71] Yang L, Zhang Y (2017). Tumor-associated macrophages: from basic research to clinical application. J Hematol Oncol.

[72] Vinay DS, Kwon BS (2014). 4-1BB (CD137), an inducible costimulatory receptor, as a specific target for cancer therapy. BMB Rep.

[73] Yin Y, Pastrana JL, Li X, Huang X, Mallilankaraman K, Choi ET, Madesh M, Wang H, Yang XF (2013). Inflammasomes: sensors of metabolic stresses for vascular inflammation. Front Biosci (Landmark Ed).

[74] Yu Y, He Y, Yang TT, Jiang H, Xiang YJ, Fang LB, Hjelmstrom P, Gao XG, Liu GZ (2014). Elevated plasma levels and monocyte-associated expression of CD137 ligand in patients with acute atherothrombotic stroke. Eur Rev Med Pharmacol Sci.

[75] Jeon HJ, Choi JH, Jung IH, Park JG, Lee MR, Lee MN, Kim B, Yoo JY, Jeong SJ, Kim DY (2010). CD137 (4-1BB) deficiency reduces atherosclerosis in hyperlipidemic mice. Circulation.

[76] Olofsson PS, Soderstrom LA, Wagsater D, Sheikine Y, Ocaya P, Lang F, Rabu C, Chen L, Rudling M, Aukrust P (2008). CD137 is expressed in human atherosclerosis and promotes development of plaque inflammation in hypercholesterolemic mice. Circulation.

[77] Dumitriu IE, Baruah P, Finlayson CJ, Loftus IM, Antunes RF, Lim P, Bunce N, Kaski JC (2012). High levels of costimulatory receptors OX40 and 4-1BB characterize CD4+CD28null T cells in patients with acute coronary syndrome. Circ Res.

[78] Srivastava RM, Trivedi S, Concha-Benavente F, Gibson SP, Reeder C, Ferrone S, Ferris RL (2017). CD137 stimulation enhances cetuximab-induced natural killer: dendritic cell priming of antitumor T-cell immunity in patients with head and neck cancer. Clin Cancer Res.

[79] Ju S, Ge Y, Qiu H, Lu B, Qiu Y, Fu J, Liu G, Wang Q, Hu Y, Shu Y (2009). A novel approach to induce human DCs from monocytes by triggering 4-1BBL reverse signaling. Int Immunol.

[80] Jung IH, Choi JH, Jin J, Jeong SJ, Jeon S, Lim C, Lee MR, Yoo JY, Sonn SK, Kim YH (2014). CD137-inducing factors from T cells and macrophages accelerate the destabilization of atherosclerotic plaques in hyperlipidemic mice. FASEB J.

[81] Gauttier V, Judor JP, Le Guen V, Cany J, Ferry N, Conchon S (2014). Agonistic anti-CD137 antibody treatment leads to antitumor response in mice with liver cancer. Int J Cancer.

[82] Webb GJ, Hirschfield GM, Lane PJ (2016). OX40, OX40L and autoimmunity: a comprehensive review. Clin Rev Allergy Immunol.

[83] Dongming L, Zuxun L, Liangjie X, Biao W, Ping Y (2010). Enhanced levels of soluble and membrane-bound CD137 levels in patients with acute coronary syndromes. Clin Chim Acta.

[84] Foks AC, van Puijvelde GH, Bot I, ter Borg MN, Habets KL, Johnson JL, Yagita H, van Berkel TJ, Kuiper J (2013). Interruption of the OX40-OX40 ligand pathway in LDL receptor-deficient mice causes regression of atherosclerosis. J Immunol.

[85] van Wanrooij EJ, van Puijvelde GH, de Vos P, Yagita H, van Berkel TJ, Kuiper J (2007). Interruption of the Tnfrsf4/Tnfsf4 (OX40/OX40L) pathway attenuates atherogenesis in low-density lipoprotein receptor-deficient mice. Arterioscler Thromb Vasc Biol.

[86] Vu MD, Xiao X, Gao W, Degauque N, Chen M, Kroemer A, Killeen N, Ishii N, Li XC (2007). OX40 costimulation turns off Foxp3+ Tregs. Blood.

[87] Yan J, Su H, Xu L, Wang C (2013). OX40-OX40L interaction promotes proliferation and activation of lymphocytes via NFATc1 in ApoE-deficient mice. PLoS One.

[88] Yu G, Li Y, Cui Z, Morris NP, Weinberg AD, Fox BA, Urba WJ, Wang L, Hu HM (2016). Combinational immunotherapy with Allo-DRibble vaccines and anti-OX40 co-stimulation leads to generation of cross-reactive effector T cells and tumor regression. Sci Rep.

[89] Weixler B, Cremonesi E, Sorge R, Muraro MG, Delko T, Nebiker CA, Daster S, Governa V, Amicarella F, Soysal SD (2015). OX40 expression enhances the prognostic significance of CD8 positive lymphocyte infiltration in colorectal cancer. Oncotarget.

[90] Gough MJ, Killeen N, Weinberg AD (2012). Targeting macrophages in the tumour environment to enhance the efficacy of alphaOX40 therapy. Immunology.

[91] Chai ZT, Zhu XD, Ao JY, Wang WQ, Gao DM, Kong J, Zhang N, Zhang YY, Ye BG, Ma DN (2015). microRNA-26a suppresses recruitment of macrophages by down-regulating macrophage colony-stimulating factor expression through the PI3K/Akt pathway in hepatocellular carcinoma. J Hematol Oncol.

[92] van de Ven K, Borst J (2015). Targeting the T-cell co-stimulatory CD27/CD70 pathway in cancer immunotherapy: rationale and potential. Immunotherapy.

[93] Winkels H, Meiler S, Smeets E, Lievens D, Engel D, Spitz C, Burger C, Rinne P, Beckers L, Dandl A (2017). CD70 limits atherosclerosis and promotes macrophage function. Thromb Haemost.

[94] van Olffen RW, de Bruin AM, Vos M, Staniszewska AD, Hamann J, van Lier RA, de Vries CJ, Nolte MA (2010). CD70-driven chronic immune activation is protective against atherosclerosis. J Innate Immun.

[95] Sardella G, De Luca L, Francavilla V, Accapezzato D, Mancone M, Sirinian MI, Fedele F, Paroli M (2007). Frequency of naturally-occurring regulatory T cells is reduced in patients with ST-segment elevation myocardial infarction. Thromb Res.

[96] Claus C, Riether C, Schurch C, Matter MS, Hilmenyuk T, Ochsenbein AF (2012). CD27 signaling increases the frequency of regulatory T cells and promotes tumor growth. Cancer Res.

[97] McEarchern JA, Oflazoglu E, Francisco L, McDonagh CF, Gordon KA, Stone I, Klussman K, Turcott E, van Rooijen N, Carter P (2007). Engineered anti-CD70 antibody with multiple effector functions exhibits in vitro and in vivo antitumor activities. Blood.

[98] Sun M, Fink PJ (2007). A new class of reverse signaling costimulators belongs to the TNF family. J Immunol.

[99] Bartlett NL, Chen R, Fanale MA, Brice P, Gopal A, Smith SE, Advani R, Matous JV, Ramchandren R, Rosenblatt JD (2014). Retreatment with brentuximab vedotin in patients with CD30-positive hematologic malignancies. J Hematol Oncol.

[100] Foks AC, Bot I, Frodermann V, de Jager SC, Ter Borg M, van Santbrink PJ, Yagita H, Kuiper J, van Puijvelde GH (2012). Interference of the CD30-CD30L pathway reduces atherosclerosis development. Arterioscler Thromb Vasc Biol.

[101] Willers J, Dummer R, Kempf W, Kundig T, Burg G, Kadin ME (2003). Proliferation of CD30+ T-helper 2 lymphoma cells can be inhibited by CD30 receptor cross-linking with recombinant CD30 ligand. Clin Cancer Res.

[102] Oflazoglu E, Stone IJ, Gordon KA, Grewal IS, van Rooijen N, Law CL, Gerber HP (2007). Macrophages contribute to the antitumor activity of the anti-CD30 antibody SGN-30. Blood.

[103] Nocentini G, Ronchetti S, Petrillo MG, Riccardi C (2012). Pharmacological modulation of GITRL/GITR system: therapeutic perspectives. Br J Pharmacol.

[104] Meiler S, Smeets E, Winkels H, Shami A, Pascutti MF, Nolte MA, Beckers L, Weber C, Gerdes N, Lutgens E (2016). Constitutive GITR activation reduces atherosclerosis by promoting regulatory CD4+ T-cell responses—brief report. Arterioscler Thromb Vasc Biol.

[105] Pedroza-Gonzalez A, Zhou G, Singh SP, Boor PP, Pan Q, Grunhagen D, de Jonge J, Tran TK, Verhoef C, JN IJ (2015). GITR engagement in combination with CTLA-4 blockade completely abrogates immunosuppression mediated by human liver tumor-derived regulatory T cells ex vivo. Oncoimmunology.

[106] Kim WJ, Bae EM, Kang YJ, Bae HU, Hong SH, Lee JY, Park JE, Kwon BS, Suk K, Lee WH (2006). Glucocorticoid-induced tumour necrosis factor receptor family related protein (GITR) mediates inflammatory activation of macrophages that can destabilize atherosclerotic plaques. Immunology.

[107] Yu N, Fu S, Xu Z, Liu Y, Hao J, Zhang A, Wang B (2016). Synergistic antitumor responses by combined GITR activation and sunitinib in metastatic renal cell carcinoma. Int J Cancer.

[108] Zhou L, Ismaili J, Stordeur P, Thielemans K, Goldman M, Pradier O (1999). Inhibition of the CD40 pathway of monocyte activation by triazolopyrimidine. Clin Immunol.

[109] Lee ME, Wang H (1999). Homocysteine and hypomethylation. A novel link to vascular disease. Trends Cardiovasc Med.

[110] Wang H, Yoshizumi M, Lai K, Tsai JC, Perrella MA, Haber E, Lee ME (1997). Inhibition of growth and p21ras methylation in vascular endothelial cells by homocysteine but not cysteine. J Biol Chem.

[111] Jamaluddin MD, Chen I, Yang F, Jiang X, Jan M, Liu X, Schafer AI, Durante W, Yang X, Wang H (2007). Homocysteine inhibits endothelial cell growth via DNA hypomethylation of the cyclin A gene. Blood.

[112] Halloran PF (2004). Immunosuppressive drugs for kidney transplantation. N Engl J Med.

[113] Smeets E, Meiler S, Lutgens E (2013). Lymphocytic tumor necrosis factor receptor superfamily co-stimulatory molecules in the pathogenesis of atherosclerosis. Curr Opin Lipidol.

[114] Mukundan L, Bishop GA, Head KZ, Zhang L, Wahl LM, Suttles J (2005). TNF receptor-associated factor 6 is an essential mediator of CD40-activated proinflammatory pathways in monocytes and macrophages. J Immunol.

[115] Sanguigni V, Ferro D, Pignatelli P, Del Ben M, Nadia T, Saliola M, Sorge R, Violi F (2005). CD40 ligand enhances monocyte tissue factor expression and thrombin generation via oxidative stress in patients with hypercholesterolemia. J Am Coll Cardiol.

[116] Yang J, Zhang L, Yu C, Yang XF, Wang H (2014). Monocyte and macrophage differentiation: circulation inflammatory monocyte as biomarker for inflammatory diseases. Biomark Res.

[117] Foley RN, Parfrey PS, Sarnak MJ (1998). Clinical epidemiology of cardiovascular disease in chronic renal disease. Am J Kidney Dis.

[118] Rogacev KS, Cremers B, Zawada AM, Seiler S, Binder N, Ege P, Grosse-Dunker G, Heisel I, Hornof F, Jeken J (2012). CD14++CD16+ monocytes independently predict cardiovascular events: a cohort study of 951 patients referred for elective coronary angiography. J Am Coll Cardiol.

