# Supplementary material for: Metabolism-associated danger signal-induced immune response and reverse immune checkpoint-activated CD40+ monocyte differentiation
Source: J Hematol Oncol. 2017 Jul 24;10:141. doi: 10.1186/s13045-017-0504-1 (PMC5525309; doi:10.1186/s13045-017-0504-1)
Supplement: Additional file 1: Table S1. — Features of innate and adaptive immunity. (DOC 29 kb) [file 13045_2017_504_MOESM1_ESM.doc]

Table S1. Features of innate and adaptive immunity.

| Features | Innate immunity | Adaptive immunity |
| --- | --- | --- |
| Acquisition Inborn | Inborn | Acquired |
| Response cells | Phagocytes | TC/BC only |
| Response time | Immediately (0-92 hours) | 3-5 days |
| Responding cell ratio | Almost all cell | 1/10^6^-10^5^ cells |
| Specificity | Ag-independent | Ag-dependent |
| Receptor | Primitive and broad PRR | highly specific TCR and BCR |
| Memory | No | Yes (memory TC) |

Innate and adaptive immunity are different by their acquisition manner, response cell/time, Ag dependence, receptor specificity, and immune memory. Abbreviations: TC, T cell; BC, B cell; TCR, T cell receptor; BCR, B cell receptor; Ag, antigen; PRR, pattern recognition receptor.
